# Intrinsic FGFR2 and Ectopic FGFR1 Signaling in the Prostate and Prostate Cancer

**DOI:** 10.3389/fgene.2019.00012

**Published:** 2019-01-30

**Authors:** Cong Wang, Ziying Liu, Yuepeng Ke, Fen Wang

**Affiliations:** ^1^School of Pharmaceutical Sciences, Wenzhou Medical University, Wenzhou, China; ^2^Institute of Biosciences and Technology, Texas A&M University, College Station, TX, United States

**Keywords:** growth factor, receptor tyrosine kinase, prostate, cancer progression, cell signaling

## Abstract

Advanced castrate-resistant prostate cancer (CRPC) is a poorly prognostic disease currently lacking effective cure. Understanding the molecular mechanism that underlies the initiation and progression of CRPC will provide new strategies for treating this deadly disease. One candidate target is the fibroblast growth factor (FGF) signaling axis. Loss of the intrinsic FGF7/FGF10-type 2 FGF receptor (FGFR2) pathway and gain of the ectopic type 1 FGF receptor (FGFR1) pathway are associated with the progression to malignancy in prostate cancer (PCa) and many other epithelial originating lesions. Although FGFR1 and FGFR2 share similar amino acid sequences and structural domains, the two transmembrane tyrosine kinases elicit distinctive, even sometime opposite signals in cells. Recent studies have revealed that the ectopic FGFR1 signaling pathway contributes to PCa progression via multiple mechanisms, including promoting tumor angiogenesis, reprogramming cancer cell metabolism, and potentiating inflammation in the tumor microenvironment. Thus, suppression of FGFR1 signaling can be an effective novel strategy to treat CRPC.

## The Prostate and Prostate Cancer

The prostate is an accessory gland of the male reproductive system, which secretes many components of semen. In Western societies, prostate cancer (PCa) is the most frequently diagnosed cancer in males and the second leading cause of cancer death. The human prostate has three distinct zones: the peripheral zone, the transition zone, and the central zone, accounting for about 5, 10, and 85% of human PCa cases, respectively (Jemal et al., 2009). The progression of PCa is a slow and multiple-step process. PCa at early stages is organ-confined and androgen-responsive. Advanced PCas, however, are frequently metastatic and castration resistant (Denmeade and Isaacs, 2002). Although androgen-deprivation and surgery are common for treating PCa at early stages, there are no effective cure for advanced PCa so far. Understanding the mechanisms underlying the onset, progression to castration resistance, and metastasis of PCa is needed for developing new diagnostic, preventive, and therapeutic approaches for patients with PCa.

**FIGURE 1:**
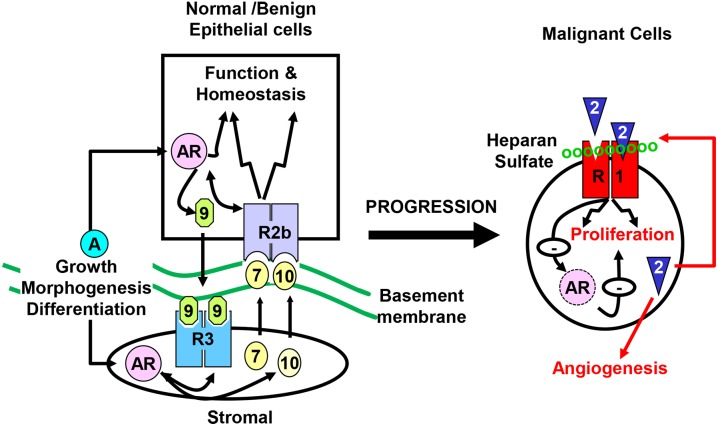
Compartmentalization of the prostate. The prostate epithelial cells are separated from the stromal cells by basement membranes. Testosterone controls direction-specific FGF signaling between the stroma and the epithelium, which maintains prostate tissue homeostasis. The paracrine FGF loops between the stroma and the epithelium is abrogated in cancer cells, and are replaced with autocrine FGF loops that promote cell proliferation, migration, survival, and angiogenesis, which contributes to castration resistance. R1, FGFR1; R2b, FGFR2IIIb; R3, FGFR3; 2, 7, 9, and 10, FGF2, FGF7, FGF9, and FGF10, respectively; AR, androgen receptor.

The prostate is composed of epithelial and stromal compartments, which are separated by basement membranes (Wang et al., 2013). The epithelium has three major cell types: luminal cells, basal cells, and neuroendocrine cells (NE) (Wang, 2011). The luminal cells express cytokeratins 8 and 18, the cell surface marker CD57, and the androgen receptor (AR). They are terminally differentiated cells that produce prostatic secretory proteins in an androgen-dependent manner. Androgen withdrawal induces massive apoptosis in the luminal cells. Basal cells, which express cytokeratins 5 and 14, CD44, and P63, reside between the luminal cells and the basement membranes. Although some basal cells weakly express the AR, many of them are AR negative and are not androgen dependent. Neuroendocrine cells, which are a minor cell population in the epithelial compartment, express synaptophysin, chromogranin A, and synaptic vesicle protein 2 (Portela-Gomes et al., 2000). The prostate stroma is a fibromuscular tissue comprised of smooth muscle (SMC)-like cells expressing α-actin and fibroblast-like cells that do not express α-actin. Although the cells in the two compartments are separated by the basement membranes, they maintain active two-way regulatory communications mediated by paracrine growth factors. The fibroblast growth factor (FGF) signaling axis is a major regulatory mechanism in the prostate, required for the development, tissue homeostasis, and function of the prostate (Figure 1). Mutations, ablations, and abnormal activations of the FGF signaling axis components are pathological and contribute to cancer development and progression. Disruption of these homeostasis-promoting two-way regulatory communications is a common feature in PCa (Yan et al., 1992, 1993; Feng et al., 1997; Matsubara et al., 1998; McKeehan et al., 1998; Lu et al., 1999; Nakano et al., 1999).

## The FGF Signaling Axis

**FIGURE 2:**
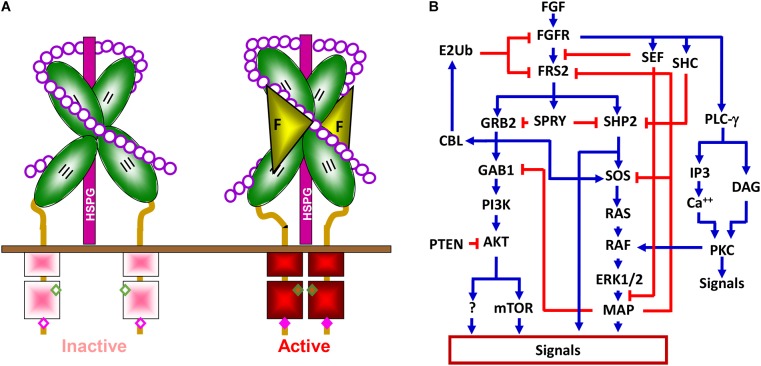
The FGFR signaling pathway. **(A)** The FGFR signaling complex. Interaction with heparin sulfate chains restricts FGFR in an inactive conformation. Docking of FGF releases this negative restriction and changes the FGFR to an active conformation where adjacent kinases trans-phosphorylate each other. II, Ig-loop II; III, Ig-loop III; F, FGF; K, kinase domain; HSPG, heparan sulfate proteoglycan; circles, monosaccharides of heparan sulfate chains; and solid bar, plasma membrane. **(B)** Simplified signaling pathways by FGFR tyrosine kinase. Blue lines represent positive regulation, and red lines represent negative effects.

The fibroblast growth factor family consists of 18 intrinsic tissue regulatory polypeptides, which controls a broad spectrum of cellular processes through binding and activating the transmembrane FGF receptor (FGFR) tyrosine kinase (Figure 2A). The FGFR is a glycosylated polypeptide that consists of an extracellular ligand-binding domain, a transmembrane domain, and an intracellular tyrosine kinase domain. Although only four genes (Fgfr1, Fgfr2, Fgfr3, and Fgfr4) encode FGFRs, the diversity of the FGFR family is substantially expanded through alternative splicing. These variants differ in their tissue distributions, binding activities and specificities for ligands and heparan sulfates, and functions. Generally, FGFR variations in the extracellular domain, particularly in the second half of Ig-like domain III, namely IIIb and IIIc, define the ligand-binding specificity, whereas in the intracellular domain, they define the signaling specificity (Li et al., 2016). In addition, heparan sulfate cofactors in the FGF-FGFR signaling complex determine not only the affinity, but also the specificity of the FGF-FGFR interaction.

Binding of the FGF to the FGFR changes the conformation of the HS-FGFR kinase complexes and leads to receptor autophosphorylation, which activates the kinase activity by altering the conformation of an auto inhibitory loop in the kinase domain. Although the four FGFRs share over 80% homology in their primary sequences, they elicit receptor- and cell-type specific activities in cells (Feng et al., 1997; Matsubara et al., 1998; Xian et al., 2007; Luo et al., 2009; Li et al., 2016; Molotkov et al., 2017). Alternative splicing of FGFR1 also contributes to signaling specificity (Brewer et al., 2015). Nevertheless, the mitogen-activated protein kinase (MAPK), phosphoinositide 3-kinase (PI3K), phospholipase Cγ (PLCγ), STAT3, P38, and JNK pathways are considered the downstream pathways in the FGFR1 signaling cascade (Brewer et al., 2016). Tyrosine phosphorylation of FGFR substrate 2α (FRS2α) by the FGFR kinase recruits the growth factor receptor-bound protein 2/son of sevenless homolog 1 (GRB2/SOS1) and SRC homology 2 domain containing phosphatase 2 (SHP2) to FGFR kinases for activation of the MAPK and PI3K/AKT signaling pathways (Figure 2B). In addition to these common pathways for FGFRs, emerging evidence demonstrates that FGFRs also have isoform-specific downstream targets, including those discussed in later sections, which, at least in part, account for FGFR signaling specificity. Future efforts are needed to identify these FGFR isoform-specific substrates or pathways.

## The Intrinsic Stroma-To-Epithelium FGF7/10-FGFR2 Signaling Axis in the Prostate Controls Prostate Development, Function, and Tissue Homeostasis

In the prostate, the epithelial and stromal compartments are separated by the basal membranes. Specific isoforms of FGF and FGFR are partitioned between the two compartments, forming directional communications between them, which regulate development, function, and tissue homeostasis of the prostate (Li et al., 2016). FGFR2 has two isoforms designated FGFR2IIIb and FGFR2IIIc as a result of highly regulated, cell type-specific, and mutually exclusive splicing of exon IIIb or exon IIIc, the two exons coding for the second half of Ig-loop 3. The expression of FGFR2IIIb and FGFR2IIIc isoforms is highly tissue specific. The exclusive expression of isoform FGFR2IIIb is a hallmark of epithelial cells in a variety of tissues that consist of multiple compartments.

Redundant FGF isoform expression governs appropriate FGF signaling in the prostate. The stromal-derived FGFs, such as FGF7 and FGF10, control epithelial cell resident FGFR2IIIb activities, promote net tissue homeostasis, and restraint tumor cells from progression to malignancy (Feng et al., 1997; Matsubara et al., 1998; Ricol et al., 1999). Interestingly, FGF10, but not FGF7, is required for prenatal, and adolescent prostate development. Ablation of the *Fgf10* alleles disrupts prenatal prostate development and the androgen-responsiveness of prostatic rudiments grafted to kidney capsules of wildtype mice (Donjacour et al., 2003).

Conditional ablation of *Fgfr2* in the prostate epithelium compromises prostate development (Lin et al., 2007). Unlike the normal prostate that composed of 4 pairs of anterior, dorsal, lateral, and ventral lobes, most *Fgfr2* null prostates only have 2 pairs of dorsal and lateral lobes with poorly formed intraluminal infoldings (Lin et al., 2007). Normal prostate undergoes significant atrophy within a few days after androgen-deprivation and fast regeneration after androgen replenishment. Intriguingly, the *Fgfr2* null prostate does not have a significant prostatic atrophy 2 weeks after castration, nor does it have significant cell proliferation after androgen replenishment to the castrated males. This indicates that the *Fgfr2* null prostate is not strictly androgen-dependent with respect to tissue homeostasis. However, similar to normal prostates, production of secretory proteins in the *Fgfr2* null prostate is strictly androgen dependent. Although the protein composition of the prostatic fluid is different between wildtype and *Fgfr2* null prostates, mice bearing *Fgfr2* null alleles are fertile, implying that ablation of *Fgfr2* in the prostate partially impairs prostate function (Lin et al., 2007). Whether other FGFR isoforms compensates the loss of FGFR2 in the prostate remains to be determined. Similarly, targeted expression of a truncated construct of FGFR2IIIb lacking the kinase domain and functions as a dominant negative FGFR2 (dnFGFR2b) in the prostatic epithelium leads to a smaller prostate in mice (Foster et al., 2002). Many epithelial prostatic ducts are disorganized and contain rounded cells that express cytokeratins and do not tightly associate with the basement membrane. The stroma compartment is also poorly organized. The smooth muscle-like cells do not form a tight layer surrounding the epithelial ducts. Together, these data demonstrate that disruption of FGFR2 signaling in the prostate epithelium compromises androgen dependency with respect to tissue homeostasis, as well as the secretory function. Therefore, it appears that the FGF7/FGF10-FGFR2 signaling axis only mediates a subset of AR signaling.

Similar to other tissues, the prostate has tissue stem cells, designated prostate stem cells (P-SCs) that are capable of giving rise to basal, luminal, and neuroendocrine cells, the three cell types in the prostate epithelium. Multiple techniques have been used to identify and characterize P-SCs, which include prostasphere or organoid cultures, renal capsule implantation, and cell lineage tracing with luminal and basal specific proteins (Bhatia-Gaur et al., 1999; Xin et al., 2007; Chua et al., 2014; Karthaus et al., 2014; Huang et al., 2015a). Both adult human and mouse prostate have two types of P-SCs: the basal cell compartment derived sphere-forming cells that express P63, designated basal P-SCs (P-bSCs), and luminal compartment derived cells that express luminal cytokeratins and NKX3.1, designated luminal P-SCs (P-lSCs) or castrate-resistant Nkx3.1-expressing cells (CARNs) (Wang et al., 2009). Ablation of *Fgfr2* in P63^+^ cells *in vitro* causes the loss of sphere-forming activity (Huang et al., 2015b). The results demonstrate that FGFR2 signaling is required for formation and maintenance of prostaspheres. Ablation of *Fgfr2* in the prostate epithelium reduces P63-expressing cells in the basal cell compartment, promotes a basal stem cell-to-luminal cell differentiation, and causes prostate developmental defects in the postnatal stage (Huang et al., 2015a,b).

Prostate cancer progression is associated with the loss of resident FGFR2b expression, which abrogates the stroma-epithelium signaling axis (Yan et al., 1993). The loss of epithelial FGFR2 and changes in HS cofactors, are often found associated with tumor progression in a variety of tissues (Wang, 2011; Wang et al., 2013; Yang et al., 2013; Li et al., 2016). In addition, expression of dnFGFR2 potentiates the development and progression of prostatic intraepithelial neoplasia (PIN) lesions induced by expression of ectopic FGFR1 kinase, demonstrating the cooperation between ablation of resident FGFR2 and expression of ectopic FGFR1 in promoting PCa progression (Jin et al., 2003; Wang et al., 2004). Restoration of FGFR2IIIb in human PC cells increases the sensitivity to chemotherapeutic reagents (Shoji et al., 2014) and in stromal cells derived from the DT3327 rat PCa model restores the interaction between PCa and prostate stromal cells (Feng et al., 1997).

## Ectopic FGF Signaling Axis Perturbs Tissue Homeostasis and Induces Tumorigenesis in the Prostate

Aberrant activation of the FGF signaling axis due to ectopic expression of FGF, FGFR, and heparan sulfate proteoglycans (HSPG) is often associated with various cancers, including PCa. The loss of the homeostasis-promoting intrinsic FGFR2 signaling and concurrent gain of ectopic FGFR1 expression in epithelial tissues of a variety of organs are found associated with tumor progression (Li et al., 2016). Ectopic expression of FGFR1IIIc appears to elicit a new set of abnormal signals in epithelial cells. At the same time, the switch from FGFR2IIIb to FGFR2IIIc isoform cuts off epithelial cells from homeostasis-promoting stromal signals (FGF7/FGF10) that activate FGFR2IIIb (Yan et al., 1993; Feng et al., 1997). Loss of FGF7/FGF10 diminishes the homeostasis promoting function of FGFR2IIIb is also common during PCa progression (Yan et al., 1992; Lu et al., 1999; Wang, 2011; Wang et al., 2013; Li et al., 2016), which, together with gain of FGF2 that activates ectopic FGFR1IIIc, contributes to PCa progression (Figure 1). Changes in HSPG core protein expression and in the sulfation patterns of heparin sulfates, which alter the FGF-FGFR specificity, are associated with cancer progression (Li et al., 2016). Multiple transgenic mouse models show that overexpression of FGF2, FGF3, FGF7, or FGF8 in prostate epithelial cells lead to prostatic lesions, ranging from low grade PIN, high-grade PIN to PCa (Chua et al., 2002; Foster et al., 2002; Song et al., 2002; Konno-Takahashi et al., 2004). Furthermore, overexpression of FGF8 promotes PCa development in Pten haploid-insufficient mice, whereas the lesions in mice carrying either the FGF8 transgene or one Pten null allele generally progress only up to PIN (Zhong et al., 2006).

FGF9 has been reported to promote PCa metastasis to bone, the major organ site of PCa metastasis. Previous studies have shown that AR-negative human PCa cells induced bone metastasis is mediated by FGF9 (Li et al., 2008) and that patients with PCa overexpressing FGF9 have a higher risk of a biochemical recurrence (Teishima et al., 2012). Overexpression of FGF9 in mouse prostate epithelial cells leads to development of prostate lesions in an expression level- and age-dependent manner (Huang et al., 2015c). Furthermore, overexpression of FGF9 in the TRAMP (transgenic adenocarcinoma of the mouse prostate) PCa model accelerates the development of advanced PCa. Interestingly, overexpression of FGF9 causes significant changes in the tumor microenvironment, which includes hyper cellularity and hyper proliferation in the stromal compartment. TGFβ1, a key signaling molecule overexpressed in reactive stroma, is expressed at a higher level in FGF9 transgenic and FGF9/TRAMP bi-genic prostates than those that do not carry the FGF9 transgene. *In vivo*, *in vitro*, and *in silico* analyses of currently available data bases all demonstrate that FGF9 promotes TGFβ1 expression (Huang et al., 2015c). Therefore, FGF9 overexpression in PCa cells augments the formation of reactive stroma in the tumor microenvironment and promotes PCa initiation and progression.

Emerging evidence shows that acquisition of ectopic FGFR1 expression in epithelial cells often accompanies with PCa progression (Wang et al., 2013). Overexpression of FGFR1 in prostate epithelial cells derived from benign, androgen-responsive Dunning 3327PAP tumors increases the malignancy of the cells in a time-dependent manner (Feng et al., 1997; Wang et al., 2002). Expression of the constitutively active FGFR1 (caFGFR1) construct in prostate epithelial cells disrupts tissue homeostasis and induces progressive PIN lesions in a time and expression level-dependent manner in transgenic mice (Jin et al., 2003; Wang et al., 2004). JOCK1, a transgenic mouse model for PCa, highly expresses the membrane-anchored FGFR1 kinase domain fused in frame with a FK506 binding protein (FKBP12) at the C-terminus (Freeman et al., 2003). Sustained activation of this FGFR kinase construct in mice by chemical induced dimerization of FK506 leads to development of low grade PIN by 12 weeks, high grade PIN by 6 months, and adenocarcinoma at later stages (Freeman et al., 2003; Acevedo et al., 2007). Androgen-deprivation to treat metastatic PCa often leads to tumors that bypass the requirement of a functional AR. These PCas are notable for increased MAPK activity. Blocking the FGFR or MAPK pathways with pharmacological inhibitors compromises the growth of these PCas both *in vitro* and *in vivo*, indicating that FGFR/MAPK contributes to escaping from the AR regulation and that the FGFR/MAPK blockade can be a new strategy to treat metastatic PCa with an AR-null phenotype (Bluemn et al., 2017).

## Ectopic FGFR1 Signaling in PCa Cells Reprograms Cell Metabolism and Promotes Tumor Growth

**FIGURE 3:**
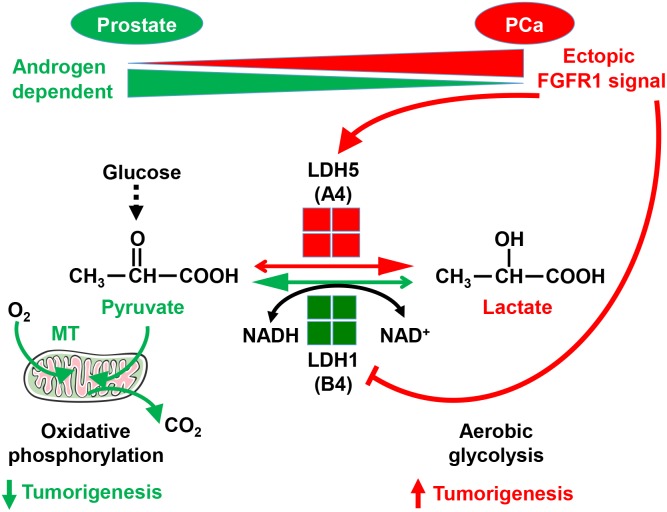
Ectopic FGFR1 signaling in reprograming cell metabolism and promoting tumorigenesis. FGFR1 reprograms cell metabolism by controlling LDH isoform expression a. A, LDHA; B, LDHB; MT, mitochondria. This figure was originally published in Liu et al. (2018).

Unlike resident FGFR2 that promotes tissue homeostasis, ectopic FGFR1 promotes tumorigenicity by stimulating proliferation and migration, as well as by preventing cell death (Li et al., 2016). We recently also reported that FGFR1 in PCa cells reprograms cellular energy metabolism by changing the expression profile of lactate dehydrogenase (LDH) isozymes (Liu et al., 2018). Metabolic reprograming from oxidative phosphorylation to aerobic glycolysis, designated the Warburg effect, is a common event in cancer progression. Compared with oxidative phosphorylation, aerobic glycolysis is less efficient with respect to generating ATP. However, it provides metabolites as building blocks for cancer cells to meet the anabolic demands of rapidly growing cells. Reduction of pyruvate to lactate is the last step of glycolysis. LDH is an enzyme catalyzing the reversible conversion between pyruvate and lactate. It is a tetramer composed of two types of subunits, LDHA, and LDHB. The LDHB subunit favors the conversion of lactate to pyruvate, and therefore, oxidative phosphorylation. The LDHA subunit, however, favors the conversion of pyruvate to lactate, and therefore, aerobic glycolysis (Fritz, 1965). FGFR1 tyrosine phosphorylates LDHA, which prevents its degradation, and therefore, increases LDHA activity in the cells. In addition, FGFR1 reduces LDHB expression by promoting CpG island methylation of the LDHB promoter (Liu et al., 2018). Through this mechanism, FGFR1 reprograms PCa cell metabolism from oxidative phosphorylation to aerobic glycolysis (Figure 3). Experiments with PCa xenografts show that LDHA depletion compromises the tumor growth, while LDHB depletion promotes tumor growth. Systematic analyses of a tissue microarray derived from PCa patients with 15 year follow-up reveal that FGFR1 overexpression is associated with high LDHA/low LDHB expression, as well as with short overall survival and biochemical recurrence times of the patients (Liu et al., 2018). The results indicate that ectopic FGFR1 expression together with LDH isoform profiles can serve as a biomarker for PCa diagnosis and prognosis.

## Ectopic FGFR1 Signaling in PCa Cells Promotes Inflammation in the Tumor Microenvironment

**FIGURE 4:**
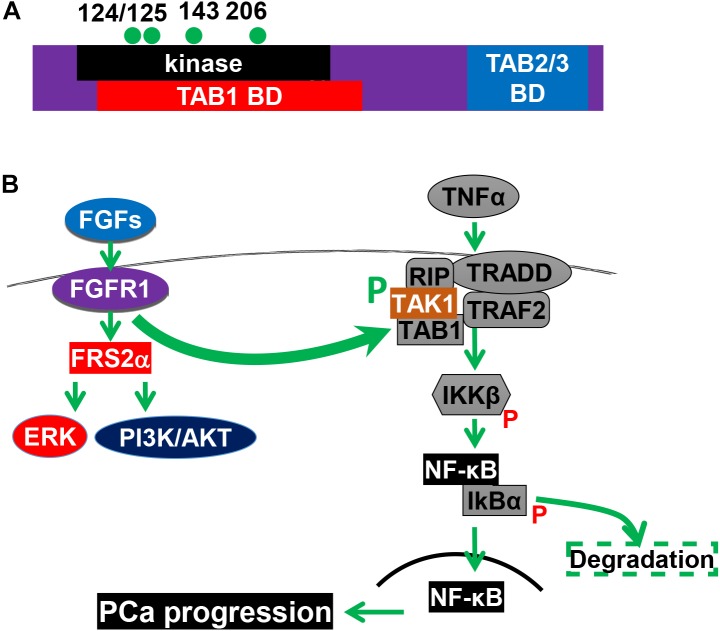
The FGFR1 pathway promotes NF-κB signaling. **(A)** Structural domains and tyrosine phosphorylation sites of TAK1. **(B)** FGFR1 phosphorylates TAK1 and augments the NF-κB pathways. 124, 125, 143, and 206, four tyrosine phosphorylation sites; BD, binding domain; CD, C-terminal binding domain; KD, kinase domain; and P, phosphorylation. This figure was originally published in Wang et al. (2018).

Inflammation in the tumor microenvironment is one of the hallmark in cancer progression (Hanahan and Weinberg, 2011). Cancer cells produce multiple inflammation-promoting chemokines and cytokines that attract lymphocytes to infiltrate into the tumor microenvironment. Among them is the NF-κB signaling axis. We recently discovered that FGF promotes NF-κB signaling in PCa cells (Wang et al., 2018). Interestingly, the three common signaling pathways downstream of FGFR1 kinase, ERK1/2, PI3K/AKT, and PLCγ, are not required for FGFR1 to augment NF-κB signaling. Instead, FGFR1 phosphorylates transforming growth factor β–activating kinase 1 (TAK1), an adaptor protein in the TNFα/NF-κB pathway. This phosphorylation reduces ubiquitination-dependent TAK1 degradation, and therefore, promotes NF-κB signaling (Figure 4). Ablation of *Fgfr1* alleles in the prostate epithelium reduces inflammation in the TRAMP tumors. Consistently, activation of the FGFR1 kinase increases inflammation in the JOCK1 mouse model (Wang et al., 2018). It has been well documented that inflammation is important for PCa initiation and progression. Therefore, promoting inflammation in the tumor microenvironment is one of the mechanisms underlying the tumor promoting activity of FGFR1.

## Inactivating Ectopic FGF Signaling Suppresses Prostate Tumorigenesis

The involvement of ectopic FGF signaling pathways in PCa initiation and progression suggests that blockading these pathways can be used to treat patients with PCa. Multiple small molecule inhibitors for the FGFR tyrosine kinases have been developed and are currently used in clinical trials for a variety of cancers, including CRPCs (Corn et al., 2013).

FGF2, which is frequently overexpressed in human PCa, promotes cell proliferation, prevents cell death, and increases tumor angiogenesis (Giri et al., 1999). Ablation of *Fgf2* alleles in TRAMP mice significantly inhibits the progression to poorly differentiated prostatic tumors, decreases metastasis to other organs, and increases the lifespan of the mice. Furthermore, ablation of only one *Fgf2* allele also affects TRAMP tumorigenesis, indicating that FGF2 has a gene dosage-dependent effect on PCa progression (Polnaszek et al., 2003). These data underscore the role of FGF2 in PCa progression as illustrated in Figure 1. TRAMP mice with *Fgfr1* ablation in prostate epithelial cells developed smaller tumors with characteristic early, well-differentiated lesions and phyllodes-type tumors. *Fgfr1* null TRAMP mice survived significantly longer than control TRAMP mice. All metastases in TRAMP/*Fgfr1* null mice were primarily those that escape *Fgfr1* disruption and highly expressed FGFR1, or neuroendocrine tumors regardless of FGFR1 status. Together, the results indicate that ectopic FGFR1 plays a critical role in the initiation, progression, and particularly metastasis of PCa (Yang et al., 2013).

FRS2 is an adaptor protein in the FGFR tyrosine kinase signaling cascade (Wang et al., 2016). It is extensively tyrosine phosphorylated by the activated FGFR kinase. The phosphorylation creates binding sites for GRB2 (Y196, Y306, Y349, Y392) that mainly link to PI3K activation, and SHP2 (Y436 and Y471) that mainly links the FGFR kinase to the MAP kinase pathway (Li et al., 2016; Wang et al., 2016). *Frs2α* is expressed almost ubiquitously in both fetal and adult tissues (McDougall et al., 2001). A rearrangement in the human Frs2α gene located in 12q15 is common in a range of tumors, suggesting that aberrant expression of FRS2α is involved in tumorigenesis. Disruption of *Frs2α* alleles in the prostate epithelium compromises the branching morphogenesis of the mouse prostate. Unlike *Fgfr2* ablation that weakens androgen dependency, ablation of *Frs2α* does not affect androgen dependency with respect to the production of secretory proteins and tissue homeostasis (Zhang et al., 2008). In the adult prostate, *Frs2α* is not expressed in luminal epithelial cells. However, it is highly expressed in TRAMP tumor epithelial cells. Conditional ablation of *Frs2α* alleles in prostate epithelial cells represses PCa initiation and progression, as evidenced by fewer and lower grade of PIN lesion foci at early stages, less advanced tumors at mid stages, and a longer lifespan of *Frs2α* conditional null TRAMP than that of parental TRAMP mice (Zhang et al., 2008).

Furthermore, analyses of a PCa tissue microarray consisting of 225 PCa samples reveals that over activation of FRS2α, cJUN, and hypoxia-inducible factor α (HIF1α) is positively correlated with blood vessel density and malignancy of human PCa (Liu et al., 2015). Ablation of *Frs2α* in mouse prostate epithelial cells reduces vascular endothelial growth factor A (VEGF-A) expression in a cJUN and HIF1α dependent manner. Depletion of FRS2α expression in human PCa cells and in MDA PCa 118b, a human PCa-derived xenograft, also suppresses tumor angiogenesis, as well as decreases bone metastasis of the tumor. Together, the result indicates that acquisition of FRS2α-mediated signaling in epithelial cells is involved in PCa initiation and progression. It also indicates that overexpressed FRS2α has the potential to serve as a biomarker for PCa diagnosis and prognosis.

## Remarks

Although the four FGFR kinases are highly homologous in their primary sequences and share similar structural domains and tyrosine phosphorylation sites, their signaling specificities are different and sometimes function in opposite directions. Although the four FGFRs share multiple common downstream pathways, they have isoform-specific downstream targets that contributes to isoform-specific signaling. Aberrant expression, activation, or inactivation of the FGF signaling pathways have been identified as culprits for diverse developmental disorders and diseases, including PCa. No clinical benefits have been reported in the five clinical trials using a broad tyrosine kinase inhibitor for multiple growth factor receptors, including FGFR1, to treat CRPC. In addition, simply treating PCa cells with pan-FGFR kinase inhibitors promotes neuroendocrine differentiation. Therefore, developing new FGFR isoform-specific inhibitors are urgently needed for treating cancers with ectopic FGFR isoform expression. Understanding the detailed molecular basis for the cell type- and receptor isoform-specific activities of the four FGFRs is essential for developing strategies to target FGFR isoform-specific signaling. Furthermore, suppression of ectopic FGFR1 signaling reduces inflammation in the tumor microenvironment and reverses metabolic reprogramming of cancer cells, both of which have profound effects on anti-cancer chemotherapies and anti-checkpoint treatment. Therefore, combination of blocking FGFR1 signals with other anti-cancer drugs shall be a promising novel strategy for treating CRPC and other cancers with ectopic FGFR1 expression.

## Author Contributions

All authors participated in reference gathering, reviewing, manuscript writing, and proofreading.

## Conflict of Interest Statement

The authors declare that the research was conducted in the absence of any commercial or financial relationships that could be construed as a potential conflict of interest.
